# Emerging Insights on Caspases in COVID-19 Pathogenesis, Sequelae, and Directed Therapies

**DOI:** 10.3389/fimmu.2022.842740

**Published:** 2022-02-21

**Authors:** Thomas A. Premeaux, Stephen T. Yeung, Zaheer Bukhari, Scott Bowler, Oral Alpan, Raavi Gupta, Lishomwa C. Ndhlovu

**Affiliations:** ^1^ Department of Medicine, Division of Infectious Diseases, Weill Cornell Medicine, New York, NY, United States; ^2^ Department of Pathology, The State University of New York (SUNY) Downstate Health Sciences University, Brooklyn, NY, United States; ^3^ Immunopathogenesis Section, Amerimmune, Fairfax, VA, United States; ^4^ Brain and Mind Research Institute, Weill Cornell Medicine, New York, NY, United States

**Keywords:** COVID-19, caspase, inflammation, apoptosis, therapeutic

## Abstract

Coronavirus disease 2019 (COVID-19) caused by the severe acute respiratory syndrome coronavirus 2 (SARS-CoV-2), remains a significant global health emergency with new variants in some cases evading current therapies and approved vaccines. COVID-19 presents with a broad spectrum of acute and long-term manifestations. Severe COVID-19 is characterized by dysregulated cytokine release profile, dysfunctional immune responses, and hypercoagulation with a high risk of progression to multi-organ failure and death. Unraveling the fundamental immunological processes underlying the clinical manifestations of COVID-19 is vital for the identification and design of more effective therapeutic interventions for individuals at the highest risk of severe outcomes. Caspases are expressed in both immune and non-immune cells and mediate inflammation and cell death, including apoptosis and pyroptosis. Here we review accumulating evidence defining the importance of the expression and activity of caspase family members following SARS-CoV-2 infection and disease. Research suggests SARS-CoV-2 infection is linked to the function of multiple caspases, both mechanistically *in vitro* as well as in observational studies of individuals with severe COVID-19, which may further the impact on disease severity. We also highlight immunological mechanisms that occur in severe COVID-19 pathology upstream and downstream of activated caspase pathways, including innate recognition receptor signaling, inflammasomes, and other multiprotein complex assembly, inflammatory mediators IL-1β and IL-18, and apoptotic and pyroptotic cell death. Finally, we illuminate discriminate and indiscriminate caspase inhibitors that have been identified for clinical use that could emerge as potential therapeutic interventions that may benefit clinical efforts to prevent or ameliorate severe COVID-19.

## Introduction

According to the World Health Organization (WHO), as of December 2021, the severe acute respiratory syndrome coronavirus 2 (SARS-CoV-2) pandemic and the cause of Coronavirus disease 2019 (COVID-19) has led to over 244 million infections and ~5 million deaths globally since the virus outbreak was first reported in 2019. SARS-CoV-2 infection we know now can result in a vast range of clinical pulmonary manifestations, from no symptoms to critical illness, which the latter could lead to extrapulmonary complications, including neurological, thromboembolic, cardiovascular, renal, gastrointestinal, hepatobiliary, endocrinologic, and dermatologic manifestations (1–3). Furthermore, unlike any other respiratory viruses, many individuals who recover from COVID-19 report lingering short term and long term persistent symptoms referred to as long-COVID or post-acute sequelae SARS-CoV-2 infection (PASC). Long-COVID can persist beyond 6 months after symptom onset and present with neurological, psychosocial, cardiothoracic, pulmonary, gastrointestinal, hematologic, and/or renal issues (4–7). The complexity of COVID-19 has been contentious in the area of therapies to combat the infection (8). Current FDA approved treatment for adults and children with COVID-19 include VEKLURY (remdesivir) and several emergency use authorizations (EUA) have been issued for several monoclonal antibodies, molnupiravir, and paxlovid (9–11). Treatment options targeting both the virus and/or host factors for the various stages and presentations of COVID-19 continue to expand and remain an area of critical need in an attempt to reduce the risk of hospitalization or death. With the advent of highly protective vaccines against SARS-Cov-2 infection the spread, disease severity, and mortality has been altered, though protection against novel variants of concern (VOCs) is proving an ongoing challenge.

Caspases are a highly conserved family of intracellular cysteine-dependent aspartate-specific proteases that primarily mediate cell death and inflammation (12–14). All caspases are constitutively expressed during homeostasis in both immune and non-immune cells as catalytically inactive zymogens that require appropriate signals to activate c-terminal protease domain (15). Caspases contain common highly conserved protein domains, such as caspase-associated recruitment domains (CARDS) and death effector domains (DEDs). Caspases have been functionally classified according to their involvement in either apoptosis or inflammation. Apoptosis is an immunologically silent and coordinated non-lytic process of dismantling and removing of damaged, infected, and aging cells. Host cellular apoptosis is thought to be a common viral infection response mechanism for restricting viral expansion. Much like apoptosis, inflammation is another initial host cell response to viral infection. Caspases that mediate inflammation facilitate the maturation of pro-interleukins by cleaving and activating their zymogen forms as well as promoting an inflammatory form of cell death called pyroptosis (16, 17). While there are also ‘outlier’ caspases defined by their role in the cell cycle and cell differentiation (18, 19), they are currently not known to be of significance in SARS-CoV-2 infection.

Evidence demonstrates that COVID-19 is an inflammatory disease mediated by a hyperactive immune response. Conventionally, SARS-CoV-2 gains cellular entry through the interaction of the spike protein receptor binding domain and host angiotensin-converting enzyme 2 (ACE2) receptor through endosomal mechanisms or TMPRSS2-mediated membrane fusion at the cell surface (20, 21), but noncanonical routes have also been identified (22–25). Nonetheless of entry mechanisms, uncoated viral RNA is released into the cell cytosol for damage recognition by host cell pattern recognition receptors (PRRs), such as Toll-like receptors (TLRs) and RIG-I like Receptors (RLRs), which can elicit a robust immune response. While SARS-CoV-2 viral RNA interactions with endosomal TLRs and RLRS can lead to the production of NF-κB pro-inflammatory cytokines (i.e. IL-6, IL-1β) and type I and III interferons (26), SARS-CoV-2 proteins can also activate host TLR2 pathways to induce pro-inflammatory cytokine production (27, 28). However, caspase activity is also a significant contributor to the pronounced cellular death and inflammatory characteristics of COVID-19. Unraveling caspase-related immunological processes contributing to COVID-19 sequelae is vital to identify and design effective host targeted therapeutic interventions for individuals at the highest risk of severe outcomes. This review, will focus on updates on the role of caspases and COVID-19 in disease pathogenesis and targeted therapies being considered to ameliorate disease outcomes.

## Caspase Pathways in Inflammation and During SARS-CoV-2 Infection

**Figure 1:**
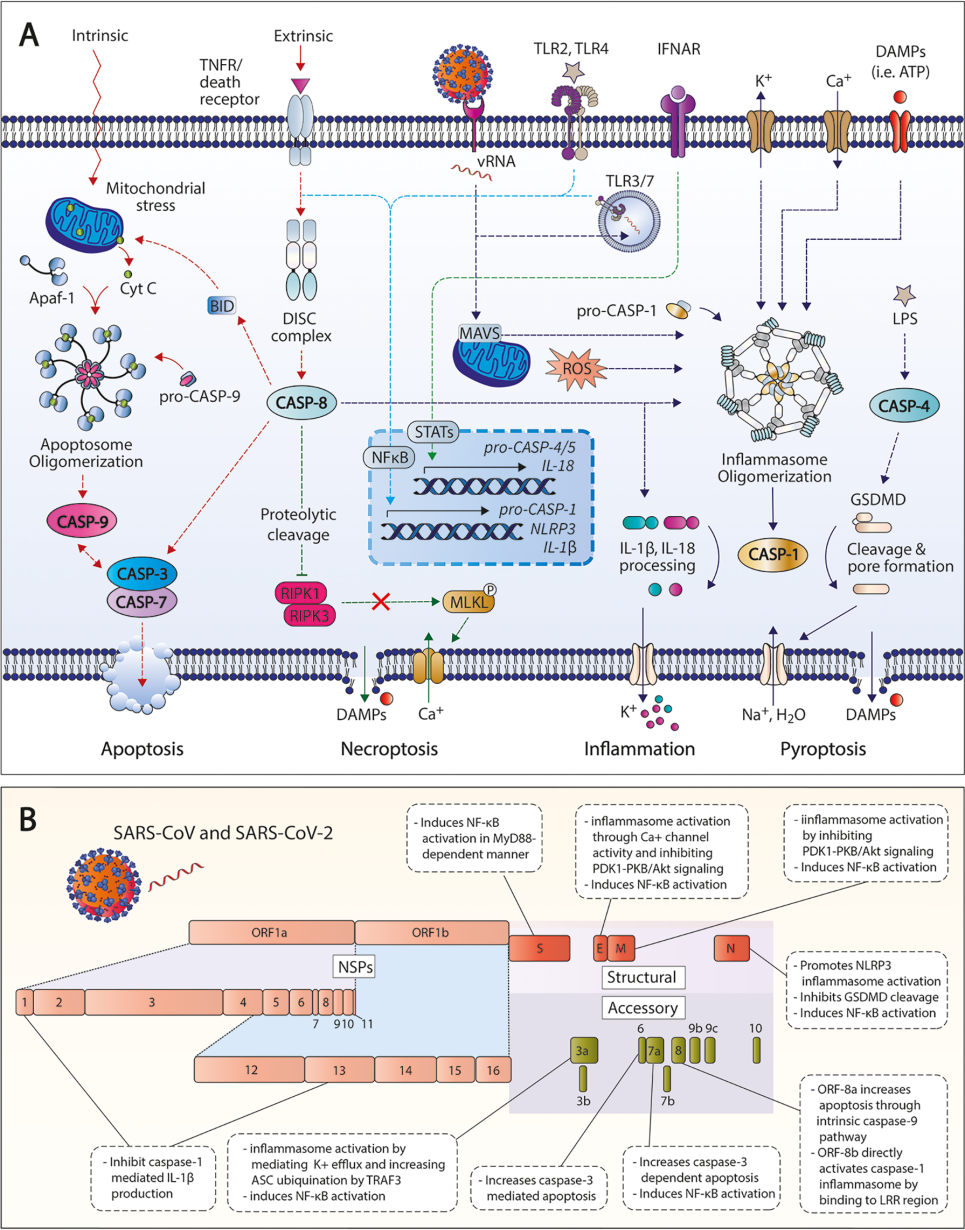
Activated caspase pathways in SARS-CoV-2. **(A)** Apoptotic and inflammatory pathways associated with caspases in SARS-CoV-2 infection and COVID-19. **(B)** Structural, non-structural, and accessory proteins of SARS-CoV and SARS-CoV-2 that modulate caspase-related pathways. TNFR, tumor necrosis factor receptor; CASP, caspase; DISC, death-inducing signaling complex; GSDMD, gasdermin D; ROS, reactive oxygen species; LDH, lactate dehydrogenase; DAMPs, danger-associated molecular patterns; TNFR, tumor necrosis factor receptor; IFNAR, interferon α/β receptor; LPS, lipopolysaccharide; RIPK, receptor-interacting serine/threonine, protein kinase; MLKL, mixed lineage kinase domain-like protein; NSP, non-structural protein.

Previous preclinical studies have suggested the role of caspases primarily as inflammatory and apoptotic mediators in various pathologies, including Inflammatory, neurological and metabolic diseases, and cancer. Accumulating evidence reveal new insights on the importance of caspase-mediated inflammatory and apoptotic pathways during SARS-CoV-2 infection (**Figure 1A**). Cell death and dysregulated caspase activation has been associated with hematological and immunological findings in patients with COVID-19 (29, 30). Like other members of the Coronaviridae family, SARS-CoV-2 is an enveloped single-stranded positive-sense RNA virus comprised of four structural proteins: nucleocapsid (N), membrane (M), envelope (E), and spike (S) proteins (31). Additionally, SARS-CoV-2 open reading frames (ORFs) also encode for various non-structural proteins (NSPs) and accessory proteins that can be involved in viral RNA transcription and replication, and/or controlling the production of other viral proteins (32). These encoded structural, non-structural, and accessory proteins can target crucial immune pathways that contribute to host immune dysregulation and active viral evasion. SARS-CoV and SARS-CoV-2 proteins shown to actively modulate the induction and/or signaling of caspase-mediated pathways are summarized in **Figure 1B**.

### Caspase-Mediated Apoptotic Pathways

Caspases that execute apoptosis either function in initiator (caspases 8, 9, and 10) or effector (caspases 3, 6, and 7) roles depending on their position in the signaling cascade (16, 17). Initiator caspases are recruited into multiprotein complexes, such as the apoptosome and death-inducing signaling complex (DISC), driven by a local increase of concentration that must be first triggered by either intrinsic or extrinsic processes. In the intrinsic pathway, intracellular stress signals lead to the release of cytochrome c (cyt c) from the mitochondria, which induces the formation of the apoptosome (33, 34). The apoptosome, consisting of cyt c and apoptotic protease-activating factor-1 (Apaf-1), recruits pro-caspase-9 *via* its N-terminal CARD. The extrinsic apoptotic pathway is mediated through the engagement of certain death receptors of the tumor necrosis factor (TNF) family (i.e. Fas), leading to the recruitment of adaptor proteins and caspases-8 or -10 into DISC *via* DED-mediated interactions (35, 36). Once recruited to multiprotein complexes, initiator caspases will dimerize to undergo proximity-induced autoactivation and act as proteolytic signal amplifiers to activate effector caspases (caspases 3, 6, and 7). However, caspase-8 can also cleave the pro-death BCL-2 family protein Bid to its truncated form (tBid) to induce cyt c release from the mitochondria and propagate the apoptotic pathway (37). Once effector caspases are activated, they induce the proteolytic degradation of multiple specific cellular substrates that facilitate the dismantling of the cell, including those that drive membrane blebbing, fragmentation of chromosomal DNA, and apoptotic body formation. Apoptosis is canonically thought of as an immunologically silent form of cell death; however, Fas-mediated apoptosis has been shown to result in the production of monocyte chemoattractant protein-1 (MCP-1), IL-6, and IL-8 (38). Furthermore, while apoptosis is considered an efficient antiviral defense to eliminate infected and damaged cells and dampen inflammation *via* the cleavage and inactivation of proinflammatory cellular signals (39), pathogen-induced apoptosis may increase infection and viral pathogenicity (40).

Several apoptotic caspases are shown to be active with SARS-CoV-2 infection. *In vitro* models using the human lung cancer line, Calu-3, found that caspases 3, 8, and 9 were cleaved into their activated forms in SARS-CoV-2-infected cells (41). Furthermore, active caspase-3 was also increased in SARS-CoV-2 infected human cortical organoids and glial cells indicating a strong link with SARS-CoV-2 inducing apoptosis (42). In COVID-19 patients, caspase-3/7 activity in red blood cells is upregulated compared to healthy individuals (43). Caspase-3 is also thought to play a role in the programmed cell death of platelets with SARS-CoV-2 infection. The internalization of SARS-CoV-2 by platelets, either *in vitro* or in COVID-19 patients, results in the colocalization of SARS-CoV-2 with phosphorylated mixed lineage kinase domain-like protein (phospho-MLKL), a mediator of necroptosis, and caspase-3 on nonpermeabilized platelets (44). This caspase-3 activity is suggested to be a potential contributor to thrombotic events observed in severe COVID-19 (45). Specific viral components of SARS-CoV-2 have been identified to modulate apoptosis *via* several mechanisms. SARS-CoV-2 accessory protein, ORF3a, was shown to induce apoptosis in Vero E6, HEK293T, and HepG2 cells *via* the extrinsic pathway, through activated caspase-8 cleavage of Bid to tBid (46). ORF-3a of SARS-CoV was previously identified to induce apoptosis through both death receptor- and mitochondria-mediated pathways, propagated through caspase 8 and 9 pathways, respectively (47–49); however, it’s pro-apoptotic capacity is shown to be greater than that of the ORF3a of SARS-CoV-2 (46). Beyond ORF-3a, SARS-CoV ORF-6, -7a, and 8a, all have been previously shown to trigger cellular apoptosis. ORF-6 induces apoptosis *via* caspase-3 mediated ER stress and JNK-dependent pathways (50), whereas ORF-8a is through a mitochondria-dependent pathway (51). While the mechanism of activation for ORF7a in promoting caspase-associated inflammation is unclear, the overexpression of ORF7a induces apoptosis in a caspase-3-dependent manner (52, 53). Finally, membrane glycoprotein M in conjunction with the N protein is also shown to trigger caspase-dependent apoptosis *via* inhibiting the activation of PDK1-PKB/Akt signaling (54).

### Caspase-Mediated Inflammation and Pyroptosis

Inflammatory caspases are recruited to their cognate activation complexes called inflammasomes, protein platforms that aggregate in the cytosol in response to different stimuli (55). However, an initial priming step is generally required mediated by NF-κB through the engagement of PPRs that recognize pathogen associated molecular patterns (PAMPs) or host-derived damage associate molecular patterns (DAMPs), such as ATP or mitochondrial DNA. The most studied inflammatory caspase, caspase-1, is engaged by inflammasomes, including the NLRP, AIM2, and IFI16 inflammasomes. Activated caspase-1 then mediates the processing and secretion of the proinflammatory cytokines IL-1β and IL-18 (56). These cytokines have multiple roles in innate immunity and in bridging adaptive immune responses. IL-18 induces downstream IFN-γ responses, while IL-1β plays roles in neutrophil influx and activation, T and B-cell activation, cytokine and antibody production, and Th17 differentiation (57–60). On the other hand, inflammatory caspases 4 and 5 directly recognize intracellular lipopolysaccharide (LPS) (61), but require an initial step through the signaling of IFNAR and subsequent members of the signal transducer and activation of transcription (STAT) protein family. Another outcome of the activation of inflammatory caspases is pyroptosis, an inflammatory-related nonprogrammed cell death driven primarily by inflammasome and caspase-4/5 mediated cleavage of the pyroptotic executor cytosolic protein gasdermin D (GSDMD) (62–64). As caspase-1-dependent cytokines and DAMPs lack secretion signals, pyroptosis is thought to be one of the prime mechanisms mediating their cellular release (65–69). Although the conventional idea that inflammatory caspase activation would be protective by enhancing immunity against SARS-CoV-2 through the removal of infected cells and recruitment of monocytes to injury sites, concomitant pyroptosis exacerbating inflammation due to cellular release of DAMPs could lead to tissue death, organ failure, and septic shock (70, 71). While caspase-8 is known predominately as a mediator of apoptosis, it is also a master regulator of pyroptosis and necroptosis (72) and is capable of processing pro-IL-1β and pro-IL-18 into their functional cytokine forms (73–75). Caspase-8 can regulate necroptosis, unregulated cell death, by preventing the phosphorylation of MLKL into its active form, phospho-MLKL, by inactivating RIPK1 and RIPK3 by proteolytic cleavage (76–78).

Excessive inflammation is central to poor clinical outcomes in COVID-19, with data suggesting caspase-mediated inflammation being an important feature. Higher levels of active caspase-1 (Casp1p20) in the sera of COVID-19 patients are associated with severe disease and poor clinical outcomes (79). Caspase-1 activity is also upregulated in CD4^+^ T cells of COVID-19 patients that were hospitalized, those with liver disease, and long-haulers (43, 80). Human caspase-4 in infected individuals and its mouse homologue caspase-11 in SARS-CoV-2 murine models were recently found to be upregulated in lung tissue histologically and promote COVID-19-associated inflammation and coagulopathy (81). SARS-CoV-2 infection activates caspase-8, which triggers inflammatory cytokine processing of pro-IL-1β in lung epithelial cells and lung cells of SARS-CoV-2-infected HFH4-hACE2 transgenic mice (41). Inflammatory mediators IL-1β and IL-18, the main cytokine products of caspase-1 activation, are observed to be increased in the lungs and sera of patients with symptomatic COVID-19 compared to asymptomatic patients and healthy individuals (82–84). IL-18 levels are also shown to correlate with other inflammatory markers in SARS-CoV-2 individuals (83). Interestingly, IL-18 can contribute to the pathology of COVID-19 by altering MAIT cell function (85). In human monocytes, caspase-1 activation along with IL-1β production and pyroptosis is observed in both SARS-CoV-2 infected *ex vivo* and from infected ICU patients (86). RNH1 protein, an inhibitor of inflammasome activation through proteasome-mediated degradation of caspase-1, is increased in the blood and lung biopsies from individuals with COVID-19 and is negatively associated with SARS-CoV-2-mediated inflammation and adverse clinical outcomes (87). *In vitro*, SARS-CoV-2 infected human monocytes demonstrate pyroptotic activity, which was associated with caspase-1 activation, IL-1β production, GSDMD cleavage, and enhanced pro-inflammatory cytokine levels (86). High serum levels of lactate dehydrogenase (LDH), an indicator of pyroptosis, is also shown to associate with poor prognosis and the extent of lung damage and disease severity in individuals with COVID-19 (88–90) and has been proposed as a potentially useful marker for monitoring treatment response in COVID-19-associated pneumonia (91).

Mechanistically, SARS-CoV-2 N protein has been shown to promote NLRP3 inflammasome activation to induce caspase-mediated inflammatory milieu (IL-1β, IL-18) and pyroptotic cell death (88, 92–94). However, N protein is also shown to inhibit the cleavage of GSDMD by caspase-1 in monocytes *in vitro* (95). In previous studies of SARS-CoV, several accessory proteins have been shown to modulate inflammasome activation. The accessory protein ORF3a is shown to act as a K+ channel to induce NLRP3 inflammasome activation (96). However, another study indicates ORF3a can promote NLRP3 inflammasome activation by enhancing the ability of TNF receptor-associated factor 3 (TRAF3) in ubiquinating the inflammasome adapter ASC (97). In macrophages, SARS-CoV ORF8b was found to directly bind the LRR of NLRP3 inflammasomes to propagate caspase-1 activation (98). However, two SARS-CoV-2 NSPs, NSP1 and NSP13, are shown to inhibit NLRP3 inflammasome caspase-1-mediated IL-1β production in the monocytic cell line THP-1 (99). The E glycoprotein of SARS-CoV is also involved in inflammasome activation, as in mouse models show that viruses lacking E protein induced lower levels of inflammasome-activated IL-1β (100) by possessing calcium ion channel activity (101). Finally, many SARS-CoV encoded proteins are shown to induce NF-κB activation *in vitro*, including ORF3a, ORF7a, M, and N proteins (102, 103).

## Therapeutic Potential of Targeting Caspase Pathways for COVID-19

The COVID-19 pandemic is going on its third year, and efforts are still converging globally to effectively distribute SARS-CoV-2 vaccinations. Global vaccination efforts have not proceeded at a similar pace worldwide and vaccine hesitancy persists in the public. Furthermore, the continuous evolution of SARS-CoV-2 could lead to new VOCs, such as the recently emerged and rapidly disseminating Omicron variant. These new VOCs could impact the efficacy of neutralizing antibodies, monoclonal or vaccine-induced, and exhibit potential for increased transmissibility, as observed with Omicron (104, 105). While vaccination and previous infection by SARS-CoV-2 so far have shown to provide protection, particularly regarding the prevention of serious disease and mortality, therapeutics are still an urgent need to attenuate severe disease and are highly investigated due to the persistent unvaccinated population, breakthrough cases, and the potential emergence of immunoevasive VOCs. Therapeutics recommended by the WHO for severe and critical COVID-19 mainly aim at disrupting the viral life cycle to limit the spread of infection, such as the use of neutralizing monoclonal antibodies (i.e. casirivimab) and the protease inhibitor Paxlovid, or to hinder the development of severe disease, including the use of systemic corticosteroids (i.e. dexamethasone). For the latter, targeting inflammatory innate immune pathways are a viable target, given the therapeutic promise of IL-6 receptor blockers, such as toclilizumab or sarilumab, in reducing severe outcomes in COVID-19 (106–109). Given the role of caspases in SARS-CoV-2, targeting related pathways could emerge as a potential therapeutic strategy that may benefit clinical efforts to prevent or ameliorate severe COVID-19.

Therapeutics for caspase-associated inflammation and cell death can be through the modulation of caspase activity directly, the targeting of upstream signaling complexes (i.e. inflammasomes), or the neutralization of caspase substrates (i.e. IL-1β). Regarding caspase targeting agents, the pan-caspase inhibitor Emricasan (EMR) was shown to attenuate caspase-1 hyperactivity in CD4^+^ T cells from COVID-19 patients *ex vivo* (43) and the caspase-8 inhibitor Z-IETD-FMK subdued SARS-CoV-2-induced BID cleavage and caspase-3 activation (41). However, direct caspase-1 inhibition did not affect SARS-CoV-2-induced IL-1β processing and secretion (41). Interestingly, several caspase inhibitors were shown to target the main protease of SARS-CoV-2 M^pro^, including pan-caspase inhibition with Z-VAD(OMe)-FMK and discriminate inhibitors Z-DEVD-FMK and Z-IETD-FMK, for caspase-3 and caspase-8, respectively (110). Furthermore, among ~6,070 drugs screened, EMR was identified to inhibit the activity of Mpro *in vitro* and through computation screening shown to bind to ACE2 (111, 112). Nonetheless, while several targeted and indiscriminate caspase inhibitors have been identified and developed with intended therapeutic use, only few have advanced into clinical trials, and none are used clinically. However, therapeutics targeting the downstream effects of caspase-mediated inflammation and pyroptosis are making progress. The use of IL-1 receptor antagonist anakinra in COVID-19 patients showed significant decreases in oxygen requirements, increased duration without invasive mechanical ventilation, and decreases of fever and C-reactive protein, indicating early IL-1 receptor blockade could hold therapeutic value in acute hyperinflammatory respiratory failure (113). The anti-IL-1β antibody inhibitor canakinumab was also suggested as a viable therapeutic for COVID-19 patients (114); however, a recent clinical trial investigating its use showed that it did not significantly increase survival without invasive mechanical ventilation (115). NLRP3 inflammasome inhibition with MCC950 reduced lung inflammation and COVID-19-like pathology in human ACE2 transgenic mice infected with SARS-CoV-2 (116). Finally, Disulfiram, the GSDMD inhibitor that covalently modifies GSDMD to block pyroptotic pore formation, was shown to associate with a lower incidence of COVID-19 in a retrospective study (117).

## Conclusion

This review highlights multiple caspases implicated in SARS-CoV-2 infection and disease severity. Although targeting caspases and related pathways may be a promising intervention, caspase signaling may still be paramount for functional and balanced immune activity against SARS-CoV-2 infection. Further understanding the roles caspase pathways play during the progression of infection and disease including PASC is crucial for further therapeutic development or the repurposing of drugs, combination therapies to curtail inflammation and cell death in COVID-19 and limit disease severity and death in all age and risk groups following SARS-CoV-2 infection.

## Author Contributions

TP and LN drafted the manuscript. TP developed the graphical figures. All authors contributed to the discussion and approved the submitted version.

## Conflict of Interest

Author OA was employed by company Amerimmune.

The remaining authors declare that the research was conducted in the absence of any commercial or financial relationships that could be construed as a potential conflict of interest.

## Publisher’s Note

All claims expressed in this article are solely those of the authors and do not necessarily represent those of their affiliated organizations, or those of the publisher, the editors and the reviewers. Any product that may be evaluated in this article, or claim that may be made by its manufacturer, is not guaranteed or endorsed by the publisher.
